# Model-Informed Drug Development for Malaria Therapeutics

**DOI:** 10.1146/annurev-pharmtox-010715-103429

**Published:** 2017-10-06

**Authors:** Kayla Ann Andrews, David Wesche, James McCarthy, Jörg J. Möhrle, Joel Tarning, Luann Phillips, Steven Kern, Thaddeus Grasela

**Affiliations:** 1 Cognigen Corporation, a subsidiary of Simulations Plus, Buffalo, New York 14221, USA; email: Kayla.Andrews@cognigencorp.com, Luann.Phillips@cognigencorp.com, grasela@cognigencorp.com; 2 Department of Pharmaceutical Sciences, State University of New York, Buffalo, New York 14214, USA; 3 Bill and Melinda Gates Foundation, Seattle, Washington 98109, USA; email: David.Wesche@gatesfoundation.org, Steven.Kern@gatesfoundation.org; 4 QIMR Berghofer Medical Research Institute, Brisbane, Australia; 5 School of Medicine, University of Queensland, Brisbane, Australia; email: j.mccarthy@uq.edu.au; 6 Medicines for Malaria Venture, Geneva 1215, Switzerland; email: moehrlej@mmv.org; 7 Mahidol-Oxford Tropical Medicine Research Unit, Faculty of Tropical Medicine, Mahidol University, Bangkok, Thailand; email: joel@tropmedres.ac; 8 Centre for Tropical Medicine and Global Health, Nuffield Department of Medicine, University of Oxford, Oxford OX3 7FZ, United Kingdom

**Keywords:** malaria, model-informed drug development, controlled human malaria infection, pharmacokinetics, pharmacodynamics, disease-drug model

## Abstract

Malaria is a critical public health problem resulting in substantial morbidity and mortality, particularly in developing countries. Owing to the development of resistance toward current therapies, novel approaches to accelerate the development efforts of new malaria therapeutics are urgently needed. There have been significant advancements in the development of in vitro and in vivo experiments that generate data used to inform decisions about the potential merit of new compounds. A comprehensive disease-drug model capable of integrating discrete data from different preclinical and clinical components would be a valuable tool across all stages of drug development. This could have an enormous impact on the otherwise slow and resource-intensive process of traditional clinical drug development.

## INTRODUCTION

The United Nations estimates that 6.2 million lives have been saved over the last 15 years owing to enhanced interventions to control malaria (1). Malaria eradication would save an estimated 11 million lives and result in US$2 trillion in economic benefit (2). Drugs, vaccines, diagnostics, surveillance techniques, and innovative vector control methods are among the tools that will enable the elimination of malaria (2).

**Figure 1:**
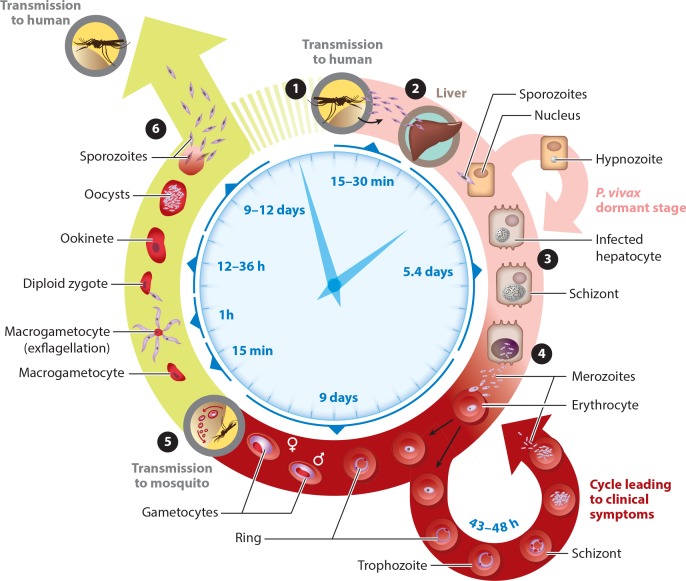
The malaria lifecycle: from mosquito to human and back. [onesansinv] Sporozoites are injected into the human bloodstream by the *Anopheles* mosquito. [twosansinv] The sporozoites travel to, and invade hepatocytes in, the liver. The infected hepatocytes mature into schizonts ([threesansinv]), which then rupture and release thousands of merozoites into the blood ([foursansinv]). Merozoites infect erythrocytes and can either enter asexual reproduction or differentiate into gametocytes. [fivesansinv] The erythrocytes containing gametocytes can then be taken up by the mosquito during a blood meal. Once the gametocytes are within the mosquito, they form sporozoites ([sixsansinv]), which can then be injected into a human host during the mosquito’s next blood meal. Modified with permission from Medicines for Malaria Venture.

Female *Anopheles* mosquitoes, whose saliva may carry one of the five species of the *Plasmodium* parasite, transmit malaria. The *Plasmodium* parasite has a complicated life cycle that includes sexual and asexual stages involving both mosquitoes and humans (**Figure 1**). Depending on the species, the parasite may also reside for a prolonged period in the host liver, resulting in relapsing malaria. *Plasmodium falciparum* is the most deadly of the five species of parasites, as it can cause severe malaria and is responsible for a majority of malaria-attributed deaths globally.

The pipeline for malaria therapeutics is growing, in part because of the tropical disease priority review vouchers offered by the US Food and Drug Administration (FDA). These vouchers have provided a large financial incentive for pharmaceutical companies to develop drugs for neglected tropical diseases and malaria (3, 4). Because most of the therapeutics will not command a price in the marketplace much beyond their cost to manufacture, there is a strong incentive for malaria therapeutic developers to use their drug development resources for this area most efficiently.

Here, we review the current state of the art in malaria drug development and describe the role of modeling and simulation in drug development and regulatory decision making. New experimental techniques continue to emerge and provide the impetus to develop improved models to predict clinical drug pharmacokinetic (PK) and pharmacodynamic (PD) properties. These new models have the potential to radically alter drug development and regulatory decision-making processes and lead to more affordable and more effective drug therapy.

**Pharmacokinetics (PK):** the study of the time course of how the body affects the drug, commonly characterized by absorption, distribution, metabolism, excretion, and toxicity (ADMET) parameters

**Pharmacodynamics (PD):** the study of how the drug affects the body, describing the relationship between drug concentrations and pharmacological response

## MALARIA DRUG DEVELOPMENT

The World Health Organization (WHO) recommends artemisinin-based combination therapies (ACTs) for the treatment of malaria. Artemisinin and its derivatives are potent and fast-acting drugs that cause a rapid decline in parasitemia during the first days of treatment. The ACTs are remarkable malaria treatments that have activity against both asexual blood-stage and sexual stages of parasite; however, the ACTs do not clear the latent liver stages of parasite*.* ACTs are required to demonstrate clinical efficacy of *>*95% polymerase chain reaction (PCR)-corrected cure rates at day 28 in the per-protocol population in clinical trials (5). ACTs are given as multiple doses over 3 days, which can lead to issues with compliance.

Unfortunately, resistance to the artemisinins, characterized by a reduced rate of parasite clearance, has arisen in the Greater Mekong Subregion of Asia, and there is concern that resistance is spreading. Decreased efficacy of artemisinins could result in exposure to the partner drug in the combination therapy at a suboptimal level in a patient with an increased parasite load. This partial treatment with less effective therapy may not completely clear parasites, allowing transmission to occur. An increased prevalence, severity, or both of artemisinin resistance would be an enormous setback in the efforts to end malaria. With this in mind, novel approaches to accelerate the development efforts of new malaria therapeutics are urgently needed.

Malaria therapy capable of achieving radical cure (i.e., eliminating all parasites from the body) is a critical component of efforts to eliminate malaria. The ideal attributes required to achieve radical cure include (*a*) the ability to block transmission of gametocytes to mosquitoes, (*b*) the ability to block transmission to or by insect vectors, (*c*) activity against hypnozoites, (*d*) activity against parasites sequestered in the liver (hepatic schizonts), and (*e*) the ability to clear the pathogenic asexual blood-stage parasites (5). Although it would be ideal to have one drug capable of achieving a radical cure, it is uncommon for one chemical entity to encompass all the necessary characteristics. Consequently, malaria therapeutics are given in combination to increase effectiveness and protect against the development of drug resistance.

**Figure 2:**
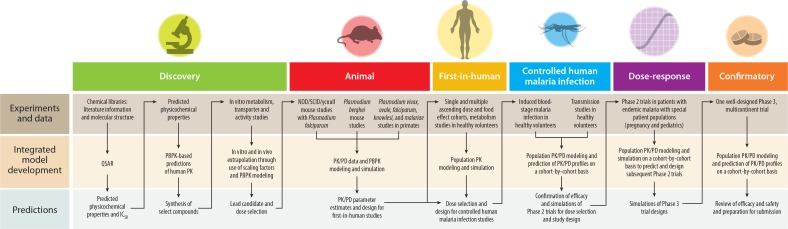
The role of modeling and simulation within each step of antimalarial research and development. Modeling and simulation translate experimental data to predictions and inform the next step in development. Abbreviations: NOD, nonobese diabetic; PBPK, physiologically based pharmacokinetic; PD, pharmacodynamics; PK, pharmacokinetics; QSAR, quantitative structure-activity relationship; SCID, severe combined immunodeficiency.

The aforementioned attributes constitute a framework of requirements that can be used to evaluate new compounds along the development path. There have been significant advancements in the preclinical in vitro and in vivo experimental models and clinical trials used to generate information used to inform decisions about the potential value of new compounds. The experimental approaches include in vitro growth of *P. falciparum* parasites, humanized severe combined immunodeficiency mice (SCID huMouse) experiments, controlled human malaria infection (CHMI) in healthy volunteers, and clinical trials in endemic settings (6–9). Altogether, these experiments have the advantage of reasonably recapitulating the human aspects of malaria infection. Nevertheless, it is difficult to compare discovery, preclinical, and clinical study characteristics directly because of differences in the experimental techniques used during the drug development process. **Figure 2** shows how experiments and data are used in sequential modeling and simulation exercises to predict the results and guide the designs of subsequent experiments in traditional model-informed drug development (MIDD). Examples of MIDD for malaria therapeutics are discussed below for discovery, preclinical and clinical stages.

**Physiologically based pharmacokinetic (PBPK) models:** quantitative prediction of pharmacokinetic properties of chemicals based on in vitro physiological, biochemical, and physicochemical properties

## DISCOVERY

The availability of large chemical libraries and high-throughput screening assays has radically altered the process of generating and selecting lead compounds. High-throughput screening assays have been successfully formatted for asexual parasites, and assays are in development to evaluate activity in both liver-stage parasites and gametocytes. Quantitative structure-activity relationship models can be used to identify the chemical structures critical to drug activity and design (e.g., impact on key physicochemical properties, such as hydrophobicity, solubility, pKa, and permeability). This information serves as input into physiologically based PK (PBPK) models and provides predictions of the absorption, distribution, metabolism, excretion, and toxicity (ADMET) characteristics of potentially useful drugs before compounds are actually synthesized (10). These PBPK models enable simulations of human drug concentration–time profiles and facilitate the selection of lead compounds for synthesis and continued assessment.

After synthesis, in vitro experiments provide additional information about the metabolic profile, transporter activity, and potency against various stages of the parasite life cycle. In vitro in vivo extrapolation (IVIVE) methods are then used to connect in vitro data to in vivo endpoints. IVIVE ranges from simple scaling factors and allometric approaches to complex mathematical models (e.g., PBPK models). These approaches are often used to predict drug-drug interactions and inform study designs in drug development (11–13).

In silico tools such as mechanistic PBPK models can be connected with a PD model in an effort to predict clinical endpoints. The value of these in silico tools was recently demonstrated by a series of experiments in which novel compounds were designed from publicly available information and were used to predict the activity, ADMET risk profiles, and in vivo PK profiles to identify lead compounds. These selected compounds were then synthesized and experimentally evaluated in vitro. The experiments showed general agreement between observed and predicted physicochemical properties, demonstrating the utility of modeling and simulation to identify potential lead compounds with acceptable PK/PD characteristics (14).

## PRECLINICAL

Unlike many disease conditions for which preclinical models may not recapitulate the actual clinical condition in patients, the experimental model of malaria infection in huMouse can be used to accrue data that help identify the target in vivo concentrations necessary for killing malaria parasites that infect humans. Experiments with different strains of parasites have been extremely useful for assessing drug activity against a variety of resistant parasite strains that are currently circulating in areas with endemic malaria. These experiments constitute a preclinical testing paradigm for malaria therapeutics that is more robust than is typically found in other therapeutic areas.

In the past, preclinical experiments to support malaria drug development were conducted in mice infected with *Plasmodium berghei* (a rodent-specific parasite species). As a result, the drug effect on *P. berghei* had to be extrapolated to represent drug activity against parasite species that infect humans. Thus, it was crucial to develop the use of immunodeficient mice to enable the study of *P. falciparum* infections in an experimental animal model. The evolution of these mouse models began with nude mice in 1962, progressed to SCID mice in 1983, and subsequently has evolved into more advanced mouse models that are capable of supporting the circulation of human erythrocytes infected with *P. falciparum* or *P. vivax* (15). The immunodeficient mouse strain NOD/SCID/γcnull (available from The Jackson Laboratory, Bar Harbor, ME) engrafted with human erythrocytes supports intravenous infections of competent *P. falciparum* strains adapted to grow in vivo and that achieve *>*10% parasitemia as measured by infected human erythrocytes (16). This malaria SCID huMouse model is currently the preferred tool for preclinical evaluation. The model has been validated using standard malaria therapeutics (e.g., chloroquine, artesunate, and pyrimethamine) and has been used successfully to characterize in vivo efficacy properties of new drug candidates with different modes of action (17–22).

**Pharmacokinetic/pharmacodynamic (PK/PD) models:** quantitative description of pharmacokinetics and pharmacodynamics to represent pharmacological relationships as a drug effect over time

**Parasite reduction ratio:** the regression-predicted fractional reduction in parasitemia at usually 48 h postinoculation

**IC_50_:** concentration of drug in blood or plasma eliciting 50% of maximum parasite killing rate

Typical preclinical experimental endpoints in the SCID huMouse model include survival, reduction in parasitemia, the effect of a compound on erythrocytic stages, time to parasite clearance, relapse, and recrudescence (23). Historically, the endpoints of preclinical experiments were focused on initial parasite clearance, and compounds were classified as either rapid acting or long duration of action rather than meeting a numerical threshold of animals cured.

As the experimental mouse model evolved, the computational models describing the data became more sophisticated. Earlier mouse experiments did not routinely include blood sampling frequencies adequate to produce the rich data needed for modeling individual PK/PD profiles. Current blood sampling schemes in the SCID huMouse model allow simultaneous monitoring of both parasitemia and drug concentrations in these animals. This information can be used to establish PK/PD relationships of potential malaria therapeutics, as has been done in the past with *P. berghei* (24–26).

Researchers have investigated the potential of the SCID huMouse model to translate estimated critical parameters to those observed in clinical trials (8). Recent studies demonstrated a poor correlation between parasite clearance rates (in vivo parasite reduction ratios) derived from mouse models and those reported from patients with malaria (8); this result is consistent with previous observations (27). However, the estimation of the IC_50_ from the SCID huMouse model has yielded promising results as a surrogate of IC_50_ in humans (8). If a valid quantitative relationship can be established that allows preclinical data to predict the parasite count over time in humans, this may facilitate forecasting of clinical outcomes when combined with PK data. If this can be validated, it places SCID huMouse models at the forefront of malaria therapeutics research, highlights their potential to provide early information about new compounds, and will help the design of safe and effective dosing regimens in clinical trials.

## CLINICAL

A drug candidate that successfully meets preclinical criteria will undergo testing in a series of clinical trials, including first-in-human (FiH), CHMI, Phase 2 dose-response, and Phase 3 confirmatory studies. The purpose of these studies is to determine the safety and efficacy profile of the drug and define the optimal treatment regimen. Data generated from each of these studies provide an opportunity for PK/PD modeling and simulation analyses to aid in the design of the subsequent studies and provide an estimate of the clinical potential of the compound.

FiH studies are conducted in healthy volunteers using single and multiple dose administrations, testing different doses and formulations in both fed and fasted states. Data obtained from these studies provide key PK information, such as drug exposure and half-life, and the identification and characterization of metabolites. The decision to advance compounds to FiH trials is based on the margin between the (*a*) estimated human efficacious dose and exposure in human and (*b*)the no observed adverse effect level (NOAEL) dose and exposure in the preclinical toxicity studies. Dose selection for FiH studies is further guided by the PD parameter estimations derived from the SCID huMouse experiments and exposure predictions from animal PK studies. A demonstrated exposure-response relationship in the SCID huMouse model increases the probability of advancing an effective drug into later-stage clinical trials. Preclinical estimations of effective concentrations, combined with the PK results from the FiH studies, can be used to validate and update PBPK models that are used in selecting the dosing regimens to be used for the CHMI studies.

**Sporozoite:** the infective stage of the parasite in mosquito saliva; is passed to the human host and ultimately infects human liver cells

**Synchronicity of parasites:** the synchronicity of the stages of the parasite in a malaria infection

CHMI studies are emerging as a tool that allows early understanding of drug activity against *Plasmodium* parasites in the human host. Two types of CHMI studies are important in malaria drug development: sporozoite-induced malaria and induced blood-stage malaria (IBSM). The sporozoite-induced malaria studies are conducted via direct venous inoculation of sporozoites or through infected *Anopheles* bites, whereas the IBSM studies are conducted via direct venous inoculation of blood-stage malaria. The pros and cons of using sporozoite-induced malaria or IBSM challenge models have been discussed (6, 9). Allan Saul and colleagues developed the IBSM experimental design in the early 1990s. These studies are typically conducted for testing vaccines, and more recently, the design has been adapted for drug studies and integrated into FiH studies. IBSM offers advantages of logistical ease in comparison to sporozoite-induced malaria studies and allows for the manipulation of inocula size.

During these IBSM CHMI studies, healthy volunteers are intravenously inoculated with synchronous parasites. In natural malaria infections, there may be some variability in the time of parasite emergence from the liver, whereas in IBSM studies, only ring-stage parasites are injected (other stages are killed by freezing). Although three *P. falciparum* strains and one *P. vivax* strain have been used, the most frequently used parasite strain is the chloroquine-sensitive *P. falciparum* clone 3D7, which is thawed from cryopreserved inocula before injection (21). Following inoculation of the parasite, the subject’s vital signs and parasite counts are monitored for safety. Once parasite counts reach the designated treatment threshold, the volunteers are treated with a single dose of experimental drug (21). Thereafter, drug concentrations and parasite counts are measured over time. Parasite counts are measured by quantitative PCR, which has a decreased lower limit of detection (just below 100 parasites/mL) compared to microscopy (approximately 10,000–100,000 parasites/mL). CHMI studies provide an opportunity to obtain key information about the PK/PD properties of a drug in a controlled setting in otherwise healthy subjects. Although these studies are absent of factors generally present in typical Phase 2 trials in infected patients (i.e., presence of multiple strains and species of parasites, comorbidities, history of disease, and natural immunity), they provide important PK/PD insights that can be directly translatable to later clinical studies to estimate an efficacious dose.

The parasite-count-over-time data from inoculation to drug dose can be used to relate observed trends in parasite growth to the parasite life cycle. The primary goal of CHMI studies is to characterize the PK/PD parameters in healthy volunteers following infection with the *Plasmodium* parasite (7). The data obtained postdose are rich in PD information and allow for examination of patterns of recrudescence and an opportunity to evaluate the characteristics of the postdose growth curve during recrudescence as compared to drug-naive parasite growth. CHMI studies are not required as part of a regulatory submission to the FDA, nor have they been included as supportive information. However, these studies are acknowledged as an important new experimental technique during development of malaria therapeutics and are increasingly being recognized and considered by the FDA (6). Researchers envision that this approach will serve as a proof of efficacy and greatly assist in the design and potential streamlining of later-stage clinical trial outcomes.

As with other drug development programs, later-stage development consists of Phase 2 dose-ranging trials and confirmatory Phase 3 trials conducted in patients with clinical malaria. Phase 2a studies focus on monotherapy, whereas Phase 2b studies investigate combination therapy. Typical Phase 2 studies often include 300–450 patients from particular geographic locations where malaria is endemic; Phase 3 studies commonly expand the scope of the patient population to include thousands of patients recruited from different geographic regions. These study populations are important because they provide the opportunity to study the determinants of response in patients who differ in body size, disease status, age, pregnancy, and immunity as well as geographic distribution.

Historically, researchers have had great difficulty in assembling and analyzing the data needed to ensure appropriate efficacy and safety for dosing special patient populations (i.e., young children and pregnant women). It took two decades to obtain enough safety data for artemetherlumefantrine to be approved as a recommendation by the WHO for women in their first trimester of pregnancy (5). Many of the currently used antimalarial drugs were also introduced at the wrong doses, especially for pregnant women and small children (28–33). This is primarily due to a lack of understanding of the pharmacological properties of the drugs used. Many physiological processes change during pregnancy, leading to altered PK properties; furthermore, physiological processes do not commonly scale linearly with body weight. These alterations in pregnant populations may result in underexposure to the antimalarial drugs when a fixed dosage (mg/kg) target is used for all patient populations. Only through PK/PD modeling of data obtained in these populations can we provide information on optimal dosing in all patients to avoid underexposure resulting in treatment failures and resistance or overexposure resulting in unnecessary dose-related adverse events. However, PBPK/PD models could be used advantageously to predict and simulate drug exposures and therapeutic outcomes of novel antimalarial drugs in these patient populations. Using specialized pediatric PBPK models and virtual neonatal, infant, and children populations, PK behavior can be modeled and simulated in these special and vulnerable populations, taking into account age-related ontogeny (34, 35). Similarly, the use of specialized pregnancy PBPK models that account for physiological and anatomical changes that occur during pregnancy allows MIDD to facilitate more efficient drug development for both maternal and pediatric populations (36).

## STRATEGIES FOR DEVELOPING A DISEASE-DRUG MODEL FOR MALARIA

The innovative experimental models that have been developed for malaria, such as the SCID huMouse model and CHMI studies, represent opportunities to integrate the otherwise sequential and separate modeling and simulation exercises to advance MIDD for malaria therapeutics. A comprehensive disease-drug model capable of integrating the separate preclinical and clinical trial components that could be used across all stages of drug development would be of great value.

MIDD uses mathematical models to quantitatively investigate the interaction of pharmacology, parasite biology, and disease pathophysiology. Models that incorporate the components of the parasite life cycle and pharmacology (37–40) as well as disease pathophysiology (41) have been extensively researched and can be used to predict the effect of drugs with different mechanisms of action at specific stages of the parasite life cycle. Mathematical constructs have also been used to describe many other aspects of the malaria infection, including but not limited to transmission dynamics (42, 43), vector control (44, 45), biological control (46), outcomes in mass drug campaigns (47), cost-effectiveness (48), drug resistance (49, 50), population genetic models (51), and development of immunity (52, 53). However, it is important to note that the true value of modeling and simulation does not lie only within specific disciplines, but rather, it is the ability to link and integrate between disciplines. Using mechanistic models and pharmacometric techniques, this becomes possible.

These mechanistic mathematical models can characterize the PK/PD properties of drugs and explore the impact of intrinsic and extrinsic factors that contribute to their intersubject variability and impact exposure and response across populations. The estimation of their impact can be used to provide a quantitative basis for optimal pharmacotherapy in humans.

Current models in use include both empirical population PK/PD models for evaluating exposure-response relationships and semimechanistic models that represent different biological aspects of the parasite life cycle and pharmacology. The PK/PD models arise from work in a variety of disciplines and differ in focus and sophistication, as outlined by Simpson and colleagues (54). The models vary structurally in terms of the number of biological parameters describing the parasite life cycle, how they represent the pharmacology of drugs, and their mechanism of action in killing the parasite. Both empirical and semimechanistic models will continue to be of value to understand parasite-host interactions and allow for refinement of dosing regimens for different subgroups of patients on a case-by-case basis for each drug of interest. These models can serve as building blocks in a tool kit for the creation of a comprehensive disease-drug model for malaria.

The data coming from SCID huMouse model and CHMI studies, in conjunction with previously published computational models, represent two ways of realizing the value of MIDD in malaria therapeutics. First, the CHMI studies are conducted on a cohort-by-cohort basis using an adaptive design. The data from the sequential cohorts of subjects enrolled in the CHMI studies provide staged learning about the PK/PD of a drug. As each cohort completes a study cycle, the parameters and structure of a PK/PD model can be updated to yield improved predictions for planning the dose of the next cohort. This iterative approach of model refinement also provides an expanding knowledge base for use in performing clinical trial simulations of future Phase 2 and Phase 3 clinical trials (K.A. Andrews, J. McCarthy, J.J. Möhrle, T.H. Grasela, S. Kern, et al., manuscript in preparation).

**Figure 3:**
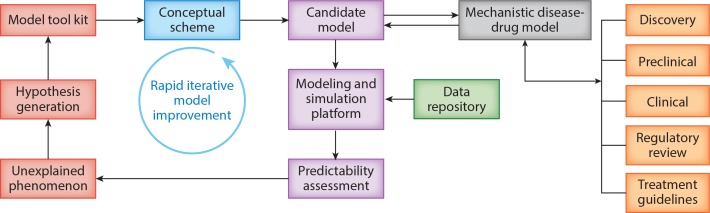
A systematic process for building, qualifying, and applying a comprehensive disease-drug model.

Second, the data that accrue from a specific drug development program, pooled with data from other programs, provide a knowledge base for the development of a comprehensive disease-drug model that is broadly applicable to drugs with differing pharmacology and across different stages of development. Building such a model will require the coordinated expertise of scientists in many disciplines. A formal process for building, qualifying, and applying a comprehensive disease-drug model is outlined in **Figure 3**.

The process of creating a comprehensive disease-drug model begins with the development of a conceptual scheme. The conceptual scheme is a pictorial representation reflecting the current understanding and interpretation of biology and pharmacology. This pictorial representation is used as the basis of reconciling different knowledge domains and points of view. As these ideas are incorporated into the conceptual scheme, the computational models can be adapted and used to perform a predictability assessment based on available data. A modeling and simulation framework can be used to integrate data and provide evidence of the validity of the model, which is important for establishing the credibility of the approach and in supporting its use for decision making during drug development and the regulatory review process. An interdisciplinary team guides this iterative process of model development, refinement, and validation. This systematic approach to model refinement, predictability assessment, hypothesis generation, and reassessment allows the strengths and weaknesses of a model to be identified and guides subsequent efforts to improve the model (55).

The predictive accuracy of any mathematical model is dependent on three sources of uncertainty: the data generated by experimental models, the validity of the mathematical construct, and how well the model represents the system of interest. Consequently, the accuracy and utility of the comprehensive disease-drug model for malaria is highly dependent on a continued assessment of the conceptual scheme and the experimental and computational models. For example, newly recognized inadequacies of the computational model at any stage of development can lead to new experimental designs that address unappreciated gaps in knowledge. This may improve the accuracy of predictions in downstream applications of the model.

## APPLICATIONS FOR A DISEASE-DRUG MODEL FOR MALARIA

The value of the model is dependent on its successful application in two different venues: at various drug development decision-making milestones and during regulatory review. Each of these venues is explored briefly below.

### Impact on Clinical Development

Federal regulations require that a new drug application must provide substantial evidence of efficacy for the investigational drug or combination of drugs in adequate and well-controlled clinical trials. Furthermore, the combination rule requires that data are available to demonstrate that each component of a fixed-dose combination contributes a measurable advantage over the individual components (e.g., increased efficacy, reduced emergence of resistance, fewer or less severe adverse events, or a simplified treatment regimen). The typical development scenario for combination therapy relies on a factorial design that compares the drug combination dose ratios to monotherapy with each separate drug to standard of care. These factorial design trials are expensive, are logistically difficult to perform, and limit the ability to deliver new malaria drug candidates in a useful time frame. They also expose a large proportion of the study population to over- and underdosing, which makes this study design questionable from a medical and ethical point of view. Leveraging dose-response data from CHMI studies and using modeling and simulation to guide trial design could decrease the number of trials necessary and increase the success of the trials conducted.

**Figure 4:**
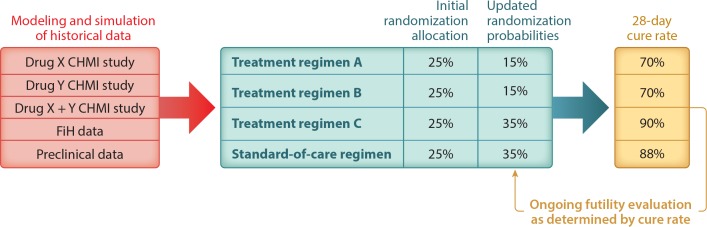
Modeling and simulation using dose-response data generated from CHMI studies guides the study design and selection of treatment regimens. The platform trial design allows for interim analysis of outcomes and updates randomization probabilities based on response to treatments. This figure shows three treatment regimens for simplicity, but there may be many more early on, as treatment regimens may represent different doses of the drugs in combination. As the quality of the predictions improves, the number of treatment regimens for various doses of Drug X and Drug Y may change. Abbreviations: CHMI, controlled human malaria infection; FiH, first-in-human.

A new study design, known as the platform trial, enables the simultaneous evaluation of multiple interventions that can be added or dropped according to accumulating real-time clinical trial information (56–58). This design provides a platform for comparing multiple experimental arms against the same control arm in a single study. Many different therapies can be evaluated, and they may be combinations or sequences of treatments. The study is designed around the idea of adapting interventions as new knowledge about subject responses emerges over the course of the trial. For example, in next-generation Phase 2 clinical trials for malaria therapies, the treatment arms might include Treatment Regimen A combination therapy, Treatment Regimen B combination therapy, Treatment Regimen C combination therapy, and standard-of-care combination therapy (as shown in **Figure 4**). The randomization probabilities to each treatment arm as well as the doses can change as knowledge about outcomes emerges through the lens of the disease-drug model. This type of pivotal platform study would pave the way to a well-designed confirmatory trial.

There are three sources of efficiency in platform trials beyond those achievable in traditional studies. First, many different types of patients can be enrolled while researchers adaptively identify and/or confirm which patient/risk factor subsets—if any—benefit from which of the therapies. Second, randomization probabilities can be modified based on the accumulating data to increase the probability of assigning better-performing therapies within a patient subtype. Finally, models of response can be built to predict long-term outcomes from short-term response data.

For malaria, this innovative study design, combined with the application of a comprehensive disease-drug model, could have a dramatic effect on the costs and probability of success of late-stage clinical trials. Clinical trial simulations could predict the outcomes of, and optimize, subsequent trial design. The models could make use of historical data to eliminate the need to replicate established exposure-response relationships or reduce the size of comparator arms. For example, dose-response information for the drugs of interest, including data from both monotherapy and combination therapy, could be obtained from CHMI studies. The PK/PD information obtained from CHMI studies can provide input to a comprehensive disease-drug model for malaria capable of representing the pharmacology of selected agents. Mathematical models could make use of these dose-response data to select rational candidates for combination therapy, guide study design for pivotal Phase 2 dose-response studies, and optimize dosing regimens. Clinical trial simulations of various scenarios could then be used to predict outcomes and optimize the likelihood of subsequent trials. Furthermore, the models would accentuate the learning during a trial and accelerate the changes in randomization probabilities to more effective treatment arms. For example, in next-generation factorial designs for malaria therapies, the treatment arms might include the standard of care, drug A treatment monotherapy, drug B treatment monotherapy, and combination therapy. The randomization probabilities to each treatment arm as well as the doses can change as knowledge about outcomes emerges through the lens of the disease-drug model.

### Impact on Regulatory Review

In 2004, the FDA launched a two-year pilot program that featured 11 stakeholder meetings and leveraged quantitative thinking to guide dose selection and clinical trial design (59). In 2009, a Guidance for Industry resulting from the FDA’s 2004–2006 pilot program outlined the purpose of End-of-Phase 2A meetings as an opportunity to “discuss options for trial designs, modeling strategies, and clinical trial simulation scenarios to improve the quantification of the exposure-response information from early drug development” (60, p. 4). The adoption and support for the use of pharmacometrics and mechanistic disease-drug models is demonstrated in the recent FDA document “Chronic Hepatitis C Virus Infection: Developing Direct-Acting Antiviral Drugs for Treatment: Guidance for Industry” (61). This guidance recommends that sponsors develop a mechanistic model of the concentration–viral kinetics and of the drug’s safety profile via pooled analysis “to predict the most active and tolerable doses to be evaluated in phase 2 trials” (61, p. 19). The review of models at the time of submission to a regulatory agency can help guide labeling recommendations.

At a recent Clinical Pharmacology Advisory Committee Meeting on MIDD hosted by the FDA, participants raised a series of issues that define the level of credibility and validity of models intended to support regulatory decision making (10). Several of these issues are directly relevant to any strategy for developing a comprehensive disease-drug model similar to what is described above. The credibility of a model reflects its relative strengths and weaknesses, including limitations of the assumptions of the model, how they can be interrogated, and where to place skepticism toward the accuracy of predictions. Model qualification should include the documentation of the timeline of model development and describe the process of improving the strengths and minimizing the weaknesses of the model. In addition, the application of the model to a specific drug development program should be supported by data-driven annotations. The robustness of the model can be demonstrated by the ability to deal with data sets arising from unique situations and the impact of the model at that time in drug development.

The Prescription Drug User Fee Act VI reauthorization provides an opportunity to assess performance goals with a view toward ensuring the effectiveness of the human drug review program and advancing MIDD concepts. The FDA has proposed a pilot program for MIDD approaches starting in fiscal year 2018; if antimalarial MIDD were explored as part of this pilot program, it would provide case studies and clarity on criteria for acceptance for various approaches to antimalarial MIDD.

SUMMARY POINTS

1. Experimental models will continue to evolve and yield additional data and insights. There is a need for valid quantitative links between the outputs of these experiments at all stages of malaria therapeutics research and development.
2. Significant advances in the experimental models used in drug development for malaria therapeutics have the potential to support more mechanistic and integrated models of malaria biology and pharmacology that could provide early estimates of the clinical potential of new antimalarial drugs.
3. The use of these models to define the exposure-response relationships has the potential to improve the selection of doses to be tested, reduce the number of patients exposed to the wrong doses, and reduce the time and resources necessary to successfully develop new antimalarial drugs.
4. Proper qualification of complex mechanistic disease-drug models and ongoing dialogue with regulatory agencies about their proper use will be required for the expanded application of MIDD for malaria therapeutics.

## DISCLOSURE STATEMENT

This work was supported by the Bill and Melinda Gates Foundation. Kayla Ann Andrews, Thaddeus Grasela, and Luann Phillips are employees of Cognigen, which was contracted, in part, by the Bill and Melinda Gates Foundation for the preparation of this manuscript. David Wesche is a consultant for the Bill and Melinda Gates Foundation through Certara Strategic Consulting. Steven Kern is a full-time employee of the Bill and Melinda Gates Foundation.
